# Gastrointestinal symptoms and health-related quality of life before and after pancreatectomy for pancreatic cancer (GO-PANC): longitudinal nationwide study

**DOI:** 10.1093/bjs/znag122

**Published:** 2026-10-06

**Authors:** Pauline A J Vissers, Annuska Schoorlemmer, Chulja J Pek, Pleun Appelhof, Lieke Baade-Corpelijn, Ellen Betten, Lisa Serlier-Erades, Henriëtte de Groot, Mariska W Hoogendoorn-de Ruiter, Sharon A G Linders, Joanne L Reeves, Annemarie M Roele-de Vreugd, Petra A W Roza-van Os, André L A Sterk, Rosa van der Werf, Marc G H Besselink, Hanneke W M van Laarhoven, Casper H J van Eijck, the Dutch Cancer Pancreatic Group, Arantza Fariña Sarasqueta, Bert A Bonsing, Ellen M Hendriksen, Gijs A Patijn, Hanneke J W Wilmink, Hjalmar C van Santvoort, Jeanin E van Hooft, Lodewijk A A Brosens, Lois A Daamen, Lydia G L M van der Geest, Mahsoem Ali, Martijn W J Stommel, Vincent E de Meijer

**Affiliations:** Department of Research and Development, Netherlands Comprehensive Cancer Organization, Utrecht, The Netherlands; Department of Surgery, Radboud University Medical Centre, Nijmegen, The Netherlands; Department of Surgery, Amsterdam UMC, location University of Amsterdam, Amsterdam, The Netherlands; Cancer Centre Amsterdam, Amsterdam, The Netherlands; Department of Surgery, Erasmus University Medical Centre, Rotterdam, The Netherlands; Department of Gastroenterology, Radboud University Medical Centre, Nijmegen, The Netherlands; Department of Surgery, Maastricht University Medical Centre, Maastricht, The Netherlands; Department of Surgery, University Medical Centre Groningen, Groningen, The Netherlands; Department of Surgery, Leiden University Medical Centre, Leiden, The Netherlands; Department of Surgery, Regional Academic Cancer Centre Utrecht, Utrecht and Nieuwegein, The Netherlands; Department of Surgery, Regional Academic Cancer Centre Utrecht, Utrecht and Nieuwegein, The Netherlands; Department of Surgery, Catharina Hospital, Eindhoven, The Netherlands; Department of Surgery, Erasmus University Medical Centre, Rotterdam, The Netherlands; Department of Surgery, Regional Academic Cancer Centre Utrecht, Utrecht and Nieuwegein, The Netherlands; Department of Surgery, Regional Academic Cancer Centre Utrecht, Utrecht and Nieuwegein, The Netherlands; Department of Surgery, Amsterdam UMC, location University of Amsterdam, Amsterdam, The Netherlands; Cancer Centre Amsterdam, Amsterdam, The Netherlands; Department of Surgery, Leiden University Medical Centre, Leiden, The Netherlands; Department of Surgery, Amsterdam UMC, location University of Amsterdam, Amsterdam, The Netherlands; Cancer Centre Amsterdam, Amsterdam, The Netherlands; Cancer Centre Amsterdam, Amsterdam, The Netherlands; Department of Medical Oncology, Amsterdam UMC, location University of Amsterdam, Amsterdam, The Netherlands; Department of Surgery, Erasmus University Medical Centre, Rotterdam, The Netherlands

## Abstract

**Background:**

Patients undergoing pancreatectomy for pancreatic cancer may experience gastrointestinal symptoms that impair health-related quality of life (HRQoL) before and after pancreatic surgery. The longitudinal course of these symptoms and their association with HRQoL remains unclear as large prospective studies are lacking.

**Methods:**

Patients who underwent pancreatectomy for pancreatic cancer and filled out questionnaires of the Dutch Pancreatic Cancer Project were included in this cohort study. Gastrointestinal symptoms and HRQoL were assessed before surgery and 0–3, 3–6, 6–9, and 9–12 months after surgery. Multivariable generalized linear mixed models were applied to evaluate longitudinal changes in gastrointestinal symptoms and their association with HRQoL.

**Results:**

After pancreatectomy for pancreatic cancer, overall, 1009 patients were included and 2995 questionnaires were completed. Before surgery, gastrointestinal symptoms were present in 95% of patients; commonly flatulence, dry mouth, problems with taste, and altered bowel habits. Gastrointestinal symptom scores peaked at 0–3 months after surgery and generally returned to preoperative or lower levels at 9–12 months after surgery. Severe gastrointestinal symptoms (defined as ‘quite a bit’ or ‘very much’) decreased from 61% of patients before surgery to 52% at 9–12 months after surgery. Patients with two or more severe symptoms had moderate (10–20 points) to large (>20 points) clinically relevant lower scores for HRQoL.

**Conclusion:**

Gastrointestinal symptoms in patients undergoing pancreatectomy for pancreatic cancer were highly prevalent and peaked within 3 months after surgery. Severe gastrointestinal symptoms affected more than half of all patients after surgery, which led to substantial declines in HRQoL.

## Teaser

Gastrointestinal symptoms in patients undergoing pancreatectomy for pancreatic cancer were highly prevalent and peaked 0–3 months after surgery. Severe gastrointestinal symptoms affected more than half of all patients after surgery, which led to substantial declines in health-related quality of life (HRQoL).

## Introduction

Pancreatic cancer (that is pancreatic ductal adenocarcinoma) is among the most lethal malignancies worldwide, with a rising incidence and poor overall survival^1^. Worldwide, the incidence of pancreatic cancer is projected to increase to nearly a million in 2050, which is an increase of 95% compared with 2022^2^. Pancreatectomy in combination with systemic therapy offers the best median survival of approximately 22 months^3^. In the Netherlands and other countries, pancreatic surgery is increasingly being centralized, resulting in decreased postoperative mortality and improved overall survival^4,5^. As survival after pancreatectomy for pancreatic cancer is increasing, more patients face the long-term consequences of their disease and treatment.

Pancreatectomy is associated with substantial morbidity, with approximately 30% of patients experiencing major complications, of which delayed gastric emptying, post-pancreatectomy haemorrhage, and postoperative pancreatic fistula are most common^5^. In addition to these complications, anatomical and functional changes in the digestive tract after resection can result in several gastrointestinal symptoms, such as nausea/vomiting, diarrhoea, and altered bowel habits^6–9^. Some retrospective studies have indicated that symptoms increase and health-related quality of life (HRQoL) scores decline after surgery, then improve thereafter^10–12^, while other studies suggest that gastrointestinal symptoms after pancreatectomy persist up to 5 years after resection^13^. These studies are often limited by small sample sizes, are single-centre and have retrospective designs, rely on cross-sectional data, or do not report on preoperative gastrointestinal symptoms, exposing critical limitations in the current evidence base.

The aim of this study was to fill this gap by evaluating self-reported gastrointestinal symptoms before and after pancreatectomy using longitudinal data from a nationwide cohort^14^. Specifically, the aim of this study was to assess both the incidence and course of gastrointestinal symptoms and their impact on HRQoL during the first year after pancreatectomy.

## Methods

### Ethics

This retrospective study was conducted with pseudonymized and aggregated data from the Netherlands Cancer Registry (NCR)/Netherlands Comprehensive Cancer Organization. Data release by the Netherlands Comprehensive Cancer Organization is subject to strict regulations, with oversight provided by a Privacy Review Board and the Scientific Committee of the Dutch Pancreatic Cancer Group (24-00459).^15^ Additional data from the Dutch Pancreatic Cancer Project (PACAP) were used. Patients provided written informed consent for participation in the PACAP and data linkage. According to Dutch legislation, this study does not fall under the scope of the Medical Research Involving Human Subjects Act (WMO) and therefore did not require approval from a medical ethics review committee.

### Data collection

Data from the PACAP and the NCR were used. The PACAP is an ongoing nationwide cohort study that collects clinical and patient-reported data to improve outcomes for patients with pancreatic cancer in the Netherlands^14^. Patients receive questionnaires at enrolment, at 3, 6, 9, 12, 18, and 24 months, and then annually thereafter. For this study, the time points were recoded to: before surgery and 0–3, 3–6, 6–9, and 9–12 months after surgery. In the case of neoadjuvant therapy, the preoperative questionnaire was filled out before, during, or after neoadjuvant chemo(radio)therapy. Clinical characteristics were obtained through linkage with the NCR. The NCR is a nationwide registry that includes all newly diagnosed malignancies in the Netherlands as reported to the automated pathological archive (PALGA) and the National Registry of Hospital Discharge Diagnoses. Trained registrars of the NCR routinely extract patient, tumour, and treatment characteristics from the medical records 9–12 months after diagnosis.

### Study population

Patients diagnosed with non-metastatic pancreatic cancer (2015–2023) who underwent pancreatectomy (that is pancreatoduodenectomy with pylorus resection (Whipple), pylorus-preserving pancreatoduodenectomy (PPPD), left pancreatectomy, or other pancreatectomies) and completed at least one questionnaire were included. Patients who solely underwent explorative surgery, without resection, were excluded.

### Patient, tumour, and treatment characteristics

Patients’ sex, age at diagnosis, ASA grade, number of co-morbidities (0, 1, or ≥2 according to a modified Charlson Co-morbidity Index), tumour location, clinical stage, radicality of resection (R1/R2 *versus* R0), type of resection, length of hospital stay after pancreatectomy, and type of (neo)adjuvant treatment were retrieved from the NCR. The anatomical site of the tumour was registered according to the International Classification of Diseases—Oncology. The Union for International Cancer Control TNM classification was used for stage, according to the version valid at the time of diagnosis (2015–2016, TNM 7th edition; and 2017–2023, TNM 8th edition). The type of resection was classified as pancreatoduodenectomy with pylorus resection (Whipple), PPPD, left pancreatectomy, or other pancreatectomies. Prolonged hospital stay was used as a proxy for postoperative complications and was defined as the 75th percentile of length of hospital stay: ≥14 days.

### Gastrointestinal symptoms and HRQoL

Gastrointestinal symptoms were assessed within the PACAP using questionnaires from the European Organisation for Research and Treatment of Cancer (EORTC), including the cancer-specific QLQ-C30 and the pancreas-specific QLQ-PAN26 modules^16,17^. From the EORTC-QLQ-C30, the scale for nausea/vomiting (2 items) was included as well as the single items of appetite loss, constipation, and diarrhoea. From the EORTC-QLQ-PAN-26, the scales for pain (4 items), eating-related problems (2 items), cachexia (2 items), and altered bowel habits (2 items) were included as well as the single items of indigestion and flatulence. In addition, the single item of ascites was included; as this term did not accurately reflect what it concerned, that is a bloated sensation, it is henceforth referred to as ‘bloated feeling’. Finally, two of the three items within the side-effects scale were included as single items, as the questions included were very diverse and will be subsequently referred to as ‘problems with taste’ and ‘dry mouth’. For a full overview of the questions included, see *Table S1*.

To assess the association between gastrointestinal symptoms and HRQoL, the functioning scales (that is physical, social, cognitive, emotional, and role functioning) and the overall quality-of-life scale from the EORTC-QLQ-C30 were included. All items were scored on a Likert scale ranging from one (not at all) to four (very much), except for the items on overall quality of life, which were scored on a scale from one (very poor) to seven (excellent). All scores were linearly transformed to the 0–100 range according to the EORTC scoring guidelines. A higher symptom score indicates more problems, whereas a higher functioning and overall quality-of-life score indicates better HRQoL.

### Statistical analysis

SAS/STAT statistical software (SAS Institute, Cary, NC, USA; version 9.4) was used for analyses.

Patient and tumour characteristics are presented. To assess survivorship bias, baseline characteristics and HRQoL were compared between patients alive and deceased at 12 months after surgery. Univariable scores for all gastrointestinal symptoms are presented and ANOVA was used to assess differences over time for each gastrointestinal symptom. To assess a possible association between (neo)adjuvant therapy and the symptoms studied, the course of all symptoms over time was stratified for patients who received neoadjuvant chemo(radio)therapy, adjuvant chemotherapy, or both neoadjuvant and adjuvant therapy. Generalized linear mixed models (GLMMs) were used to assess changes in gastrointestinal symptoms over time with questionnaire data nested within patients. These GLMMs were adjusted for age, sex, ASA grade, number of co-morbidities, tumour location, cT, cN, radicality of resection, type of resection, prolonged hospital stay, and (neo)adjuvant therapy. (Neo)adjuvant systemic therapies were modelled as time-varying covariates and were set to one only for questionnaires dated after the date of initiation of the respective systemic therapy. Additionally, severe symptoms (defined as ‘quite a bit’ or ‘very much’) were summed and categorized as zero symptoms, one symptom, or two or more symptoms, as has been done previously^18^. The number of severe gastrointestinal symptoms was subsequently summed and categorized. The proportion of severe gastrointestinal symptoms was assessed over time. Finally, the association between the number of severe gastrointestinal symptoms and HRQoL was assessed with GLMMs, with adjustment for the same variables as described above. To assess the influence of survivorship bias, both GLMMs addressing the course of symptoms over time and the association between the number of severe symptoms and HRQoL were repeated for patients who survived 12 months after surgery. Missing values were not imputed. *P* values <0.050 were regarded as statistically different. Clinical relevance was categorized as small (5–10 points), medium (10–20 points), or large (>20 points), according to Osoba *et al*.^19^.

## Results

Overall, 1009 patients treated with pancreatectomy were included, totalling 2995 completed questionnaires. Around 59% of the study population were aged ≥65 years, 55% were male, the majority (51%) had no co-morbidity, and 23% of patients died within the first year after surgery (*Table 1*). The tumour was most often located in the head of the pancreas (77%), staged cT1 or T2 (71%), and cN0 (82%). Pancreatoduodenectomy with pylorus resection (49%) or PPPD (30%) was most often performed, and around 86% of patients received neoadjuvant and/or adjuvant chemo(radio)therapy. Finally, 25% of the study population had a prolonged hospital stay (≥14 days) after resection. Of the study population, 17%, 18%, 24%, 32%, and 9% filled out one, two, three, four, and five questionnaires respectively. Patients alive at 12 months after surgery more often had an R0 resection (52% *versus* 40%), more often received adjuvant chemotherapy (65% *versus* 38%), and less often had a prolonged hospital stay (23% *versus* 33%) compared with patients who were deceased at 12 months after surgery. See *Table S2*. HRQoL was similar between both groups.

**Table 1:** Baseline characteristics of 1009 patients treated with pancreatectomy for pancreatic cancer

Characteristic	Value
**Age (years)**	
<65	418 (41)
≥65–<70	186 (18)
≥70–<75	218 (22)
≥75	187 (19)
**Sex**	
Male	550 (55)
Female	459 (46)
**ASA grade**	
I	43 (4)
II	591 (59)
III–IV	341 (34)
Unknown	34 (3)
**Number of co-morbidities**	
No co-morbidity	510 (51)
Single co-morbidity	308 (31)
Two or more co-morbidities	168 (17)
Unknown	23 (2)
**Vital status**	
Alive at 3 months after surgery	976 (97)
Alive at 6 months after surgery	929 (92)
Alive at 9 months after surgery	867 (86)
Alive at 12 months after surgery	780 (77)
**Tumour location**	
Head	774 (77)
Body	64 (6)
Tail	128 (13)
Other/unspecified	43 (4)
**cT**	
cT1	171 (17)
cT2	541 (54)
cT3	115 (11)
cT4	178 (18)
cTX	4 (0)
**cN**	
cN0	823 (82)
cN+	162 (16)
cNX	24 (2)
**Radicality**	
R0	501 (50)
R1/R2	497 (49)
Unknown	11 (1)
**Type of resection**	
PPPD	298 (30)
Pancreatoduodenectomy with pylorus resection	489 (49)
Left pancreatectomy	184 (18)
Other pancreatectomies	38 (4)
**Chemo(radio)therapy**	
None	137 (14)
Neoadjuvant	281 (28)
Adjuvant	355 (35)
Both	236 (23)
**Neoadjuvant chemotherapy**	
No	622 (62)
Yes	387 (38)
**Neoadjuvant chemoradiotherapy**	
No	879 (87)
Yes	130 (13)
**Adjuvant chemotherapy**	
No	419 (42)
Yes	590 (59)
**Prolonged hospital stay**	
No	754 (75)
Yes	255 (25)

Values are *n* (%). PPPD, pylorus-preserving pancreatoduodenectomy.

### Gastrointestinal symptoms

Before surgery, gastrointestinal symptoms (that is symptoms scored as ‘a little’, quite a bit’, or ‘very much’) were present in 95% of patients (results not shown). Flatulence, altered bowel habits, problems with taste, and dry mouth were the most common (*Fig. 1a–m*). Scores for all gastrointestinal symptoms changed significantly over time (all *P* values <0.001). In general, at 0–3 months, most symptoms increased, with small or moderate clinically relevant differences, except for nausea/vomiting and constipation. Thereafter, most symptoms decreased, resulting in comparable or lower scores at 9–12 months after surgery compared with baseline, although 94% of patients still reported at least one symptom. When stratifying univariable scores for receipt of (neo)adjuvant therapy (*Fig. S1*), patients who received neoadjuvant chemo(radio)therapy generally scored highest for diarrhoea, while patients who received adjuvant therapy reported most problems with taste after surgery. The course of gastrointestinal symptoms over time was similar for different studied subgroups (*Fig. S1*). In multivariable analyses, similar patterns were found (*Table 2*), with all symptoms, except for nausea/vomiting and constipation, increasing at 0–3 months after surgery. Scores for constipation, diarrhoea, eating-related problems, altered bowel habits, bloated feeling, indigestion, cachexia, and flatulence showed no clinically relevant differences compared with baseline at 9–12 months after surgery. At 9–12 months after surgery, pain scores were higher than preoperative scores (small clinically relevant difference). Nausea/vomiting, appetite loss, problems with taste, and dry mouth had lower scores at 9–12 months after surgery as compared with baseline (appetite loss and problems with taste: moderate clinically relevant differences; nausea/vomiting and dry mouth: small clinically relevant differences). Adjuvant chemotherapy was associated with lower scores for pain and bloated feeling and higher scores for problems with taste (all small clinically relevant differences; *Table S3*). Compared with patients who underwent PPPD, patients who underwent left pancreatectomy reported lower scores for appetite loss, altered bowel habits, and problems with taste (all small or moderate clinically relevant differences) and more constipation (small clinically relevant difference). When restricting the analyses to the 780 patients who were alive at 12 months after surgery, the increase in bloated feeling at 3–6 months and in pain at 12 months after surgery compared with baseline disappeared, for the other symptoms all observed patterns were similar and adjusted mean scores were of similar magnitude (*Table S4*).

**Figure 1:**
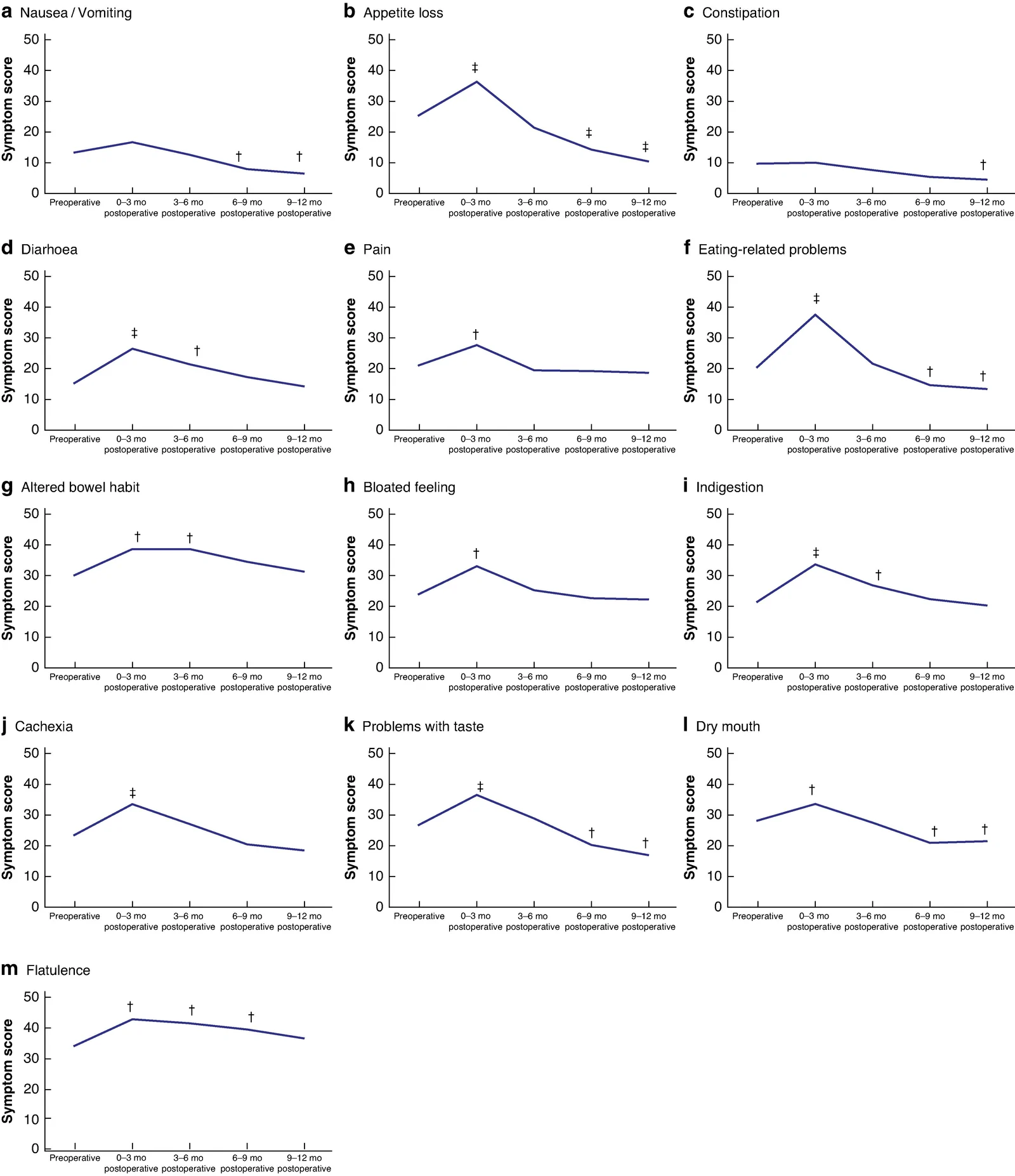
Gastrointestinal symptoms over time after pancreatectomy for pancreatic cancer †Small and ‡medium clinically relevant differences compared with preoperative scores. mo, months.

**Table 2:** Multivariable GLMMs, assessing the association of gastrointestinal symptoms over time

Gastrointestinal symptom	Preoperative	0–3 months after resection	3–6 months after resection	6–9 months after resection	9–12 months after resection
Nausea/vomiting	14.76(2.58)	18.07(2.56)*	13.68(2.53)	8.94(2.50)*†	7.07(2.54)*†
Appetite loss	23.95(3.91)	35.67(3.88)*‡	20.27(3.84)*	14.42(3.81)*†	9.36(3.83)*‡
Constipation	11.25(2.65)	11.92(2.63)	9.68(2.61)	8.12(2.58)*	7.01(2.60)*
Diarrhoea	13.88(3.71)	26.02(3.70)*‡	21.36(3.66)*†	17.92(3.64)*	15.36(3.64)
Pain	18.64(2.99)	28.35(2.97)*†	22.35(2.95)*	22.56(2.94)*	23.65(2.99)*†
Eating-related problems	20.15(3.69)	38.93(3.67)*‡	24.13(3.63)*	18.25(3.60)	16.38(3.67)*
Altered bowel habits	26.10(3.75)	35.10(3.68)*†	35.93(3.68)*†	32.15(3.67)*†	29.31(3.71)
Bloated feeling	18.80(4.04)	29.95(4.01)*‡	24.04(3.99)*†	21.94(3.97)	22.63(4.02)*
Indigestion	18.19(4.13)	31.57(4.10)*‡	25.60(4.07)*†	22.30(4.05)*	21.02(4.11)
Cachexia	19.87(3.52)	30.74(3.48)*‡	24.67(3.47)*	19.07(3.45)	16.63(3.49)*
Problems with taste	31.66(4.29)	39.53(4.24)*†	29.58(4.21)	21.66(4.19)*‡	15.81(4.25)*‡
Dry mouth	32.02(4.36)	38.04(4.30)*†	31.43(4.28)	25.27(4.26)*†	24.48(4.31)*†
Flatulence	33.97(4.23)	42.33(4.17)*†	39.82(4.18)*†	38.29(4.16)*	35.47(4.21)

Values are adjusted mean(s.e.). All gastrointestinal symptom scores range between 0 and 100, with a higher score referring to more problems. Analyses adjusted for age, sex, ASA grade, co-morbidity, location of the tumour, cT, cN, radicality, type of surgery, (neo)adjuvant chemo(radio)therapy, and length of hospital stay. Full models are presented in *Table S2*. *Significant difference compared with baseline. †Small clinically relevant difference compared with baseline. ‡Moderate clinically relevant difference compared with baseline. GLMMs, generalized linear mixed models.

### Number of severe gastrointestinal symptoms and the association with HRQoL

Before surgery, severe gastrointestinal symptoms (that is symptoms scored as ‘quite a bit’ or ‘very much’) were present in 61% of patients, which increased to 73% at 0–3 months after surgery and then decreased to 52% at 9–12 months after surgery (*Fig. 2*; *P* < 0.001). At 9–12 months after surgery, 22% of patients reported having one severe gastrointestinal symptom, and 30% of patients reported having two or more severe gastrointestinal symptoms. Similar to all symptoms, altered bowel habits, problems with taste, dry mouth, and flatulence were the most common severe symptoms.

**Figure 2:**
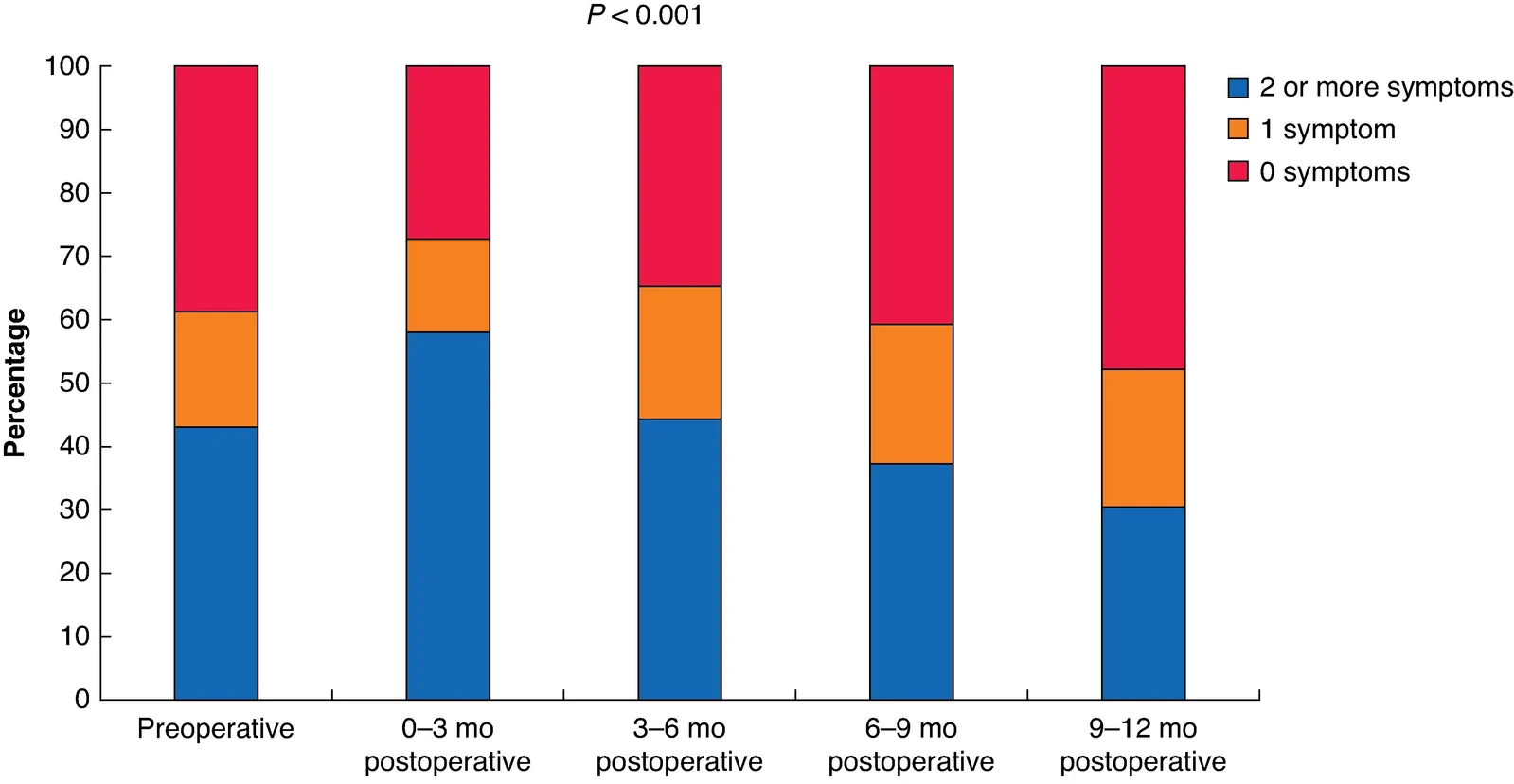
Number of severe gastrointestinal symptoms (defined as ‘quite a bit’ or ‘very much’) over time categorized as zero symptoms, one symptom, or two or more symptoms mo, months.

Multivariable analyses showed that having one severe gastrointestinal symptom or two or more severe gastrointestinal symptoms was significantly associated with quality of life (*Table 3*). Having one severe gastrointestinal symptom *versus* zero severe gastrointestinal symptoms was associated with a small clinically relevant decrease in global health status (74.6 *versus* 80.5), and role functioning (71.3 *versus* 78.6). Having two or more severe gastrointestinal symptoms *versus* zero severe gastrointestinal symptoms was associated with a moderate clinically relevant decrease in global health status (61.9 *versus* 80.5), cognitive functioning (75.3 *versus* 85.9), emotional functioning (76.5 *versus* 87.3), physical functioning (68.2 *versus* 81.9), and social functioning (67.7 *versus* 87.4), and a large (that is >20 points) clinically relevant decrease in role functioning (53.9 *versus* 78.6). In the subgroup for patients who were alive at 12 months after surgery, similar results were found (*Table S5*).

**Table 3:** Association between the number of severe gastrointestinal symptoms and HRQoL using multivariable GLMMs

Outcome	0 severe gastrointestinal symptoms	1 severe gastrointestinal symptom	2 or more severe gastrointestinal symptoms
Global health status	80.50(2.48)	74.61(2.51)*†	61.89(2.46)*‡
Cognitive functioning	85.86(2.96)	82.65(2.97)*	75.34(2.94)*‡
Emotional functioning	87.30(2.89)	84.42(2.91)*	76.45(2.87)*‡
Physical functioning	81.92(2.63)	77.56(2.65)*	68.18(2.62)*‡
Role functioning	78.60(3.53)	71.32(3.57)*†	53.91(3.50)*§
Social functioning	87.36(3.07)	82.69(3.10)*	67.71(3.04)*‡

Values are adjusted mean(s.e.). Analyses adjusted for age, sex, ASA grade, co-morbidity, location of the tumour, cT, cN, radicality, type of surgery, (neo)adjuvant chemo(radio)therapy, and length of hospital stay. *Significant difference compared with group with zero severe gastrointestinal symptoms. †Small clinically relevant difference compared with group with zero severe gastrointestinal symptoms. ‡Moderate clinically relevant difference compared with group with zero severe gastrointestinal symptoms. §Large clinically relevant difference compared with group with zero severe gastrointestinal symptoms. HRQoL, health-related quality of life; GLMMs, generalized linear mixed models.

## Discussion

This study shows that gastrointestinal symptoms are highly frequent after pancreatectomy, although scores are similar or lower at 9–12 months after surgery compared with preoperative scores. In addition, these symptoms significantly affect HRQoL, particularly when multiple severe symptoms are present. These findings can be used in clinical practice to better inform patients, manage expectations regarding postoperative symptom trajectories, and implement structured symptom monitoring and timely intervention aimed at reducing symptom burden and improving HRQoL.

The study demonstrates that gastrointestinal symptoms are highly prevalent among patients undergoing pancreatectomy for pancreatic cancer (around 95% throughout the study interval). Gastrointestinal symptom scores peaked within 3 months after resection and decreased at 9–12 months with similar or lower scores as compared with before surgery. The most frequently reported symptoms included altered bowel habits, problems with taste, flatulence, and dry mouth, while severe symptoms remained present in more than half of patients 9–12 months after surgery. Importantly, patients with two or more severe gastrointestinal symptoms reported moderate-to-large declines in global health status and functioning. These findings indicate that gastrointestinal symptom burden is not only common but also clinically meaningful, resulting in clear implications for the perioperative trajectory.

To the authors’ knowledge, this is the first large nationwide longitudinal study to comprehensively assess gastrointestinal symptoms and their impact on HRQoL during the first year after pancreatectomy for pancreatic cancer. Previous studies have primarily examined the incidence of single symptoms and HRQoL separately after, rather than before, pancreatectomy. Several retrospective studies have suggested that symptoms and functional impairment peak immediately after surgery and decline over time^10–12,20–22^, similar to the results of the present study. However, the majority of these studies lack preoperative scores^13,20,21^ or are single-centre studies with relatively small sample sizes^11–13,20^. Moreover, several studies indicate that patients experience persistently lower HRQoL and more symptoms after pancreatectomy as compared with the general population^23–25^. Although the literature on the association between specific symptoms and HRQoL is limited, a previous study found that a composite gastrointestinal symptom score was independently associated with general quality of life^23^, consistent with the findings of the present study.

Unlike prior studies that focused on individual symptoms or overall HRQoL, the findings of the present study show a clear cumulative association between severe gastrointestinal symptoms and reduced HRQoL after pancreatectomy. Even a single severe gastrointestinal symptom was associated with clinically relevant declines in overall health and role functioning, while two or more severe symptoms resulted in moderate-to-large impairments across multiple domains. The observed graded relationship is biologically and clinically plausible. For instance, pancreatic exocrine insufficiency, commonly underdiagnosed and present in up to 49% of long-term survivors after pancreatoduodenectomy^26,27^, can cause maldigestion of fat, resulting in nutrient deficiencies, steatorrhoea, and weight loss^28^. This adds to the anatomical and functional changes in the digestive tract after resection and underlines the importance of optimizing pancreatic enzyme replacement therapy, as well as early involvement of a nutritionist to prevent or mitigate nutritional deterioration. The significant impact on HRQoL observed has important clinical implications. Patients with multiple severe symptoms may benefit from closer follow-up, proactive symptom management, and early referral to supportive or palliative care. Moreover, shared decision-making is especially important as resection is the only curative option for pancreatic cancer, but still has limited survival benefit^3^. Given the lack of an alternative treatment option, it is important to understand patients’ values and expectations regarding their treatment and its outcomes, not only regarding prognosis but also regarding symptoms and HRQoL. These results contribute to insights that support the individual trade-off between HRQoL and longevity.

Patients who underwent left pancreatectomy reported less appetite loss, altered bowel habits, and problems with taste and more constipation than patients who underwent PPPD. These results are consistent with two previous studies^12,20^, although the literature remains inconclusive^29^. The lower rate of gastrointestinal symptoms after left pancreatectomy could be explained by the lower surgical complexity and preserving the gastrointestinal continuity as compared with pancreatoduodenectomy, resulting in lower morbidity and mortality^30,31^. While these findings may support preoperative counselling and expectation management regarding symptoms, the results of the present study demonstrate considerable heterogeneity in postoperative recovery, with 22% of patients reporting one severe symptom and 30% of patients reporting two or more severe symptoms at 9–12 months after surgery, indicating that symptom trajectories should be discussed on a patient-specific basis.

In the Netherlands, follow-up care after pancreatic surgery is typically provided by surgeons and nurse practitioners, with structured visits focused on symptom management and monitoring signs of recurrence. Although guidelines exist on the management of gastrointestinal symptoms after cancer treatment^32,33^, previous studies have shown persistent underdiagnosis and undertreatment of gastrointestinal dysfunction after pancreatectomy and referral for nutritional or supportive care is not consistently implemented^20^. The findings of the present study highlight the need for evidence-based guidelines and routine symptom management for timely intervention and referral in order to prevent the escalation of symptom burden and preserve HRQoL. Moreover, prehabilitation programmes may offer opportunities to optimize patients before surgery. Ongoing studies, such as the PROMISE-P study (clinicaltrials.gov ID NCT05851534), will further clarify the role of a patient-tailored prehabilitation programme in improving postoperative outcomes.

The results of this study should be interpreted considering some limitations. First, the authors had no detailed information on postoperative complications, but a prolonged hospital stay was used as a proxy. Second, selection bias may have occurred, as, in general, younger, less frail patients with a better prognosis tend to respond more to questionnaires and are less likely to drop out^34^. This potentially leads to an underestimation of symptom burden. Moreover, the authors included a subanalysis for patients alive at 12 months after surgery, with results similar to those for the total study population. Third, information on cancer recurrence was unavailable; thus reported gastrointestinal symptoms after resection could reflect recurrence. Fourth, no information regarding management by a nutritionist, treatment of gastrointestinal symptoms, pancreatic exocrine insufficiency, or use of pancreatic enzyme replacement therapy was available. The main strength is the large nationwide cohort of patients with pancreatic cancer with longitudinal data on gastrointestinal symptoms. This adds relevant insights to the currently limited literature on this topic.

## Collaborators

Arantza Fariña Sarasqueta (Amsterdam UMC, location University of Amsterdam, Amsterdam, The Netherlands); Bert A. Bonsing (Leiden University Medical Centre, Leiden, The Netherlands); Ellen M. Hendriksen (Medisch Spectrum Twente, Enschede, The Netherlands); Gijs A. Patijn (Isala, Zwolle, The Netherlands); Hanneke J. W. Wilmink (Amsterdam UMC, location University of Amsterdam, Amsterdam, The Netherlands); Hjalmar C. van Santvoort (Regional Academic Cancer Centre Utrecht, location Utrecht and Nieuwegein, The Netherlands); Jeanin E. van Hooft (Leiden University Medical Centre, Leiden, The Netherlands, Judith M. P. G. M. de Vos-Geelen (Maastricht University Medical Centre, Maastricht, The Netherlands); Lodewijk A. A. Brosens (Regional Academic Cancer Centre Utrecht, location Utrecht and Nieuwegein, The Netherlands); Lois A. Daamen (Regional Academic Cancer Centre Utrecht, location Utrecht and Nieuwegein, The Netherlands); Lydia G. L. M. van der Geest (Netherlands Comprehensive Cancer Organization (IKNL), Utrecht, The Netherlands); Mahsoem Ali (Amsterdam UMC, location University of Amsterdam, Amsterdam, The Netherlands); Martijn W. J. Stommel (Radboud University Medical Centre, Nijmegen, The Netherlands); Vincent E. de Meijer (University Medical Centre Groningen, Groningen. The Netherlands).

## Supplementary Material

znag122_Supplementary_Data

## Data Availability

The data that support this study’s findings are available from the Netherlands Comprehensive Cancer Organization (IKNL). Data are used under license for this study and therefore not publicly available. (Analytical) code(s) are available upon (reasonable) request in consultation with the corresponding author.

## References

[1] Zottl J, Sebesta CG, Tomosel E, Sebesta M-C, Sebesta C. Unraveling the burden of pancreatic cancer in the 21st century: trends in incidence, mortality, survival, and key contributing factors. Cancers (Basel) 2025;17:1607 10.3390/cancers17101607 PMC12110279 40427106

[2] Danpanichkul P, Pang Y, Tothanarungroj P, Dejvajara D, Kim D, Saokhieo P et al Gastrointestinal cancer statistics in 2022 and projection to 2050: GLOBOCAN estimates across 185 countries. Cancer 2026;132:e70245 10.1002/cncr.70245 41468237

[3] Latenstein AEJ, van der Geest LGM, Bonsing BA, Groot Koerkamp B, Haj Mohammad N, de Hingh IHJT et al Nationwide trends in incidence, treatment and survival of pancreatic ductal adenocarcinoma. Eur J Cancer 2020;125:83–93 10.1016/j.ejca.2019.11.002 31841792

[4] Latenstein AEJ, Mackay TM, van der Geest LGM, van Eijck CHJ, de Meijer VE, Stommel MWJ et al Effect of centralization and regionalization of pancreatic surgery on resection rates and survival. Br J Surg 2021;108:826–833 10.1093/bjs/znaa146 33738473

[5] Suurmeijer JA, Henry AC, Bonsing BA, Bosscha K, van Dam RM, van Eijck CH et al Outcome of pancreatic surgery during the first 6 years of a mandatory audit within the Dutch Pancreatic Cancer Group. Ann Surg 2023;278:260–266 10.1097/SLA.0000000000005628 35866656

[6] Capurso G, Traini M, Piciucchi M, Signoretti M, Arcidiacono PG. Exocrine pancreatic insufficiency: prevalence, diagnosis, and management. Clin Exp Gastroenterol 2019;12:129–139 10.2147/CEG.S168266 PMC6432881 30962702

[7] Hendriks TE, Strijbos BTM, Francken MFG, Ali M, Suurmeijer JA, Dijkgraaf MGW et al Impact of feeding strategy after pancreatoduodenectomy on delayed gastric emptying and hospital stay: nationwide study. BJS Open 2025;9:zraf068 10.1093/bjsopen/zraf068 PMC12163990 40512076

[8] Ponz de L, Pisani R, Altieri G, Stasio RC, Lazzano P, Reni M et al Gastrointestinal symptoms in the journey of pancreatic cancer patients. Expert Rev Anticancer Ther 2025;25:883–900 10.1080/14737140.2025.2517888 40489240

[9] Scholten L, Stoop TF, Del Chiaro M, Busch OR, van Eijck C, Molenaar IQ et al Systematic review of functional outcome and quality of life after total pancreatectomy. Br J Surg 2019;106:1735–1746 10.1002/bjs.11296 PMC6899549 31502658

[10] Gehrels AM, Vissers PAJ, Sprangers MAG, Fijnheer NM, Pijnappel EN, van der Geest LG et al Longitudinal health-related quality of life in patients with pancreatic cancer stratified by treatment: a nationwide cohort study. EClinicalMedicine 2025;80:103068 10.1016/j.eclinm.2024.103068 PMC11788757 39902316

[11] Laitinen I, Sand J, Peromaa P, Nordback I, Laukkarinen J. Quality of life in patients with pancreatic ductal adenocarcinoma undergoing pancreaticoduodenectomy. Pancreatology 2017;17:445–450 10.1016/j.pan.2017.02.013 28274687

[12] Shin YC, Han Y, Kim E, Kwon W, Kim H, Jang J-Y. Effects of pancreatectomy on nutritional state, pancreatic function, and quality of life over 5 years of follow up. J Hepatobiliary Pancreat Sci 2022;29:1175–1184 10.1002/jhbp.861 33175467

[13] Shaw K, Thomas AS, Rosario VL, Sugahara KN, Schrope BA, Chabot JA et al Long-term quality of life and global health following pancreatic surgery for benign and malignant pathologies. Surgery 2021;170:917–924 10.1016/j.surg.2021.03.023 33892953

[14] Coebergh van den Braak RRJ, van Rijssen LB, van Kleef JJ, Vink GR, Berbee M, van Berge Henegouwen MI et al Nationwide comprehensive gastro-intestinal cancer cohorts: the 3P initiative. Acta Oncol 2018;57:195–202 10.1080/0284186X.2017.1346381 28723307

[15] Strijker M, Mackay TM, Bonsing BA, Bruno MJ, van Eijck CHJ, de Hingh IHJT et al Establishing and coordinating a nationwide multidisciplinary study group: lessons learned by the Dutch Pancreatic Cancer Group. Ann Surg 2020;271:e102–e104 10.1097/SLA.0000000000003779 32084037

[16] Aaronson NK, Ahmedzai S, Bergman B, Bullinger M, Cull A, Duez NJ et al The European Organization for Research and Treatment of Cancer QLQ-C30: a quality-of-life instrument for use in international clinical trials in oncology. J Natl Cancer Inst 1993;85:365–376 10.1093/jnci/85.5.365 8433390

[17] Fitzsimmons D, Johnson CD, George S, Payne S, Sandberg AA, Bassi C et al Development of a disease specific quality of life (QoL) questionnaire module to supplement the EORTC core cancer QoL questionnaire, the QLQ-C30 in patients with pancreatic cancer. EORTC Study Group on Quality of Life. Eur J Cancer 1999;35:939–941 10.1016/s0959-8049(99)00047-7 10533475

[18] van Erning FN, Nieuwenhuijzen GAP, van Laarhoven HWM, Rosman C, Gisbertz SS, Heisterkamp J et al Gastrointestinal symptoms after resection of esophagogastric cancer: a longitudinal study on their incidence and impact on patient-reported outcomes. Ann Surg Oncol 2023;30:8203–8215 10.1245/s10434-023-13952-z 37523120

[19] Osoba D, Rodrigues G, Myles J, Zee B, Pater J. Interpreting the significance of changes in health-related quality-of-life scores. J Clin Oncol 2023;41:5345–5350 10.1200/JCO.22.02776 38056079

[20] Bromley-Dulfano R, August AT, Li AY, Park W, Visser B. Characterizing gastrointestinal dysfunction after pancreatic resection: a single-center retrospective study. BMC Gastroenterol 2022;22:488 10.1186/s12876-022-02565-7 PMC9701409 36435757

[21] Li S-Z, Zhen T-T, Wu Y, Wang M, Qin T-T, Zhang H et al Quality of life after pancreatic surgery. World J Gastroenterol 2024;30:943–955 10.3748/wjg.v30.i8.943 PMC10950648 38516249

[22] Toms C, Steffens D, Yeo D, Pulitano C, Sandroussi C. Quality of life instruments and trajectories after pancreatic cancer resection: a systematic review. Pancreas 2021;50:1137–1153 10.1097/MPA.0000000000001896 34714277

[23] Allen CJ, Yakoub D, Macedo FI, Dosch AR, Brosch J, Dudeja V et al Long-term quality of life and gastrointestinal functional outcomes after pancreaticoduodenectomy. Ann Surg 2018;268:657–664 10.1097/SLA.0000000000002962 PMC7962861 30199443

[24] Fong ZV, Sekigami Y, Qadan M, Fernandez-Del Castillo C, Warshaw AL, Lillemoe KD et al Assessment of the long-term impact of pancreatoduodenectomy on health-related quality of life using the EORTC QLQ-PAN26 module. Ann Surg Oncol 2021;28:4216–4224 10.1245/s10434-021-09853-8 33774773

[25] Galouzis N, Khawam M, Alexander EV, Mesropyan L, Luu C, Khreiss MR et al Quality of life and social health in patients after pancreatic surgery. J Gastrointest Surg 2025;29:101969 10.1016/j.gassur.2025.101969 39848313

[26] Beger HG, Poch B, Mayer B, Siech M. New onset of diabetes and pancreatic exocrine insufficiency after pancreaticoduodenectomy for benign and malignant tumors: a systematic review and meta-analysis of long-term results. Ann Surg 2018;267:259–270 10.1097/SLA.0000000000002422 28834847

[27] Lan X, Robin G, Kasnik J, Wong G, Abdel-Rahman O. Challenges in diagnosis and treatment of pancreatic exocrine insufficiency among patients with pancreatic ductal adenocarcinoma. Cancers (Basel) 2023;15:1331 10.3390/cancers15041331 PMC9953920 36831673

[28] Tran TC, van Lanschot JJ, Bruno MJ, van Eijck CH. Functional changes after pancreatoduodenectomy: diagnosis and treatment. Pancreatology 2009;9:729–737 10.1159/000264638 20090394

[29] Tran Cao HS, Cloyd JM, Petzel MQB, Denbo JW, Parker NH, Nogueras-González GM et al Impact of pancreatectomy on long-term patient-reported symptoms and quality of life in recurrence-free survivors of pancreatic and periampullary neoplasms. J Surg Oncol 2017;115:144–150 10.1002/jso.24499 PMC11849053 27859270

[30] de Graaff MR, Hendriks TE, Wouters M, Nielen M, de Hingh I, Koerkamp BG et al Assessing quality of hepato-pancreato-biliary surgery: nationwide benchmarking. Br J Surg 2024;111:znae119 10.1093/bjs/znae119 PMC11095128 38747683

[31] Wilson J, Talukder MH, Somasundar P, Ahmad A. Emerging technologies for pancreas resection. R I Med J 2025;108:24–28 40561239

[32] Andreyev HJN, Muls AC, Shaw C, Jackson RR, Gee C, Vyoral S et al Guide to managing persistent upper gastrointestinal symptoms during and after treatment for cancer. Frontline Gastroenterol 2017;8:295–323 10.1136/flgastro-2016-100714 PMC5641845 29067157

[33] Muls AC, Watson L, Shaw C, Andreyev HJN. Managing gastrointestinal symptoms after cancer treatment: a practical approach for gastroenterologists. Frontline Gastroenterol 2013;4:57–68 10.1136/flgastro-2012-100218 PMC5369780 28839701

[34] de Rooij BH, Ezendam NPM, Mols F, Vissers PAJ, Thong MSY, Vlooswijk CCP et al Cancer survivors not participating in observational patient-reported outcome studies have a lower survival compared to participants: the population-based PROFILES registry. Qual Life Res 2018;27:3313–3324. 10.1007/s11136-018-1979-0 PMC6244797 30167937

