# Supplementary material for: Gastrointestinal symptoms and health-related quality of life before and after pancreatectomy for pancreatic cancer (GO-PANC): longitudinal nationwide study
Source: Br J Surg. 2026 Oct 6;113(10):znag122. doi: 10.1093/bjs/znag122 (PMC13641176; doi:10.1093/bjs/znag122)
Supplement: Supplementary file 1 [file znag122_supplementary_data.docx]

**Gastrointestinal symptoms and health-related quality of life before and after pancreatectomy for pancreatic cancer (GO-PANC): a longitudinal nationwide study**

Pauline A.J. Vissers, PhD^1,2*^, Annuska Schoorlemmer^3,4*^, Chulja J. Pek^5*^, Pleun Appelhof^6^, Lieke Baade-Corpelijn^7^, Ellen Betten^8^, Lisa Serlier-Erades^9^, Henriëtte de Groot^10,11^, Mariska W. Hoogendoorn- de Ruiter^10,11^, Sharon A.G. Linders^12^, Joanne Reeves^5^, Annemarie M. Roele-de Vreugd^10^, Petra A.W. Roza- van Os^10, 11^, André L.A. Sterk^3,4^, Rosa van der Werf^9^, Marc G.H. Besselink, MD, PhD^3,4**^, Hanneke W.M. van Laarhoven, MD, PhD^4,13**^, Casper H.J. van Eijck, MD, PhD^5**^ for the Dutch Pancreatic Cancer Group
**Shared first authorship, **Shared last authorship*

^1^Netherlands Comprehensive Cancer Organization, Department of Research and Development, Utrecht, the Netherlands
^2^Radboud University Medical Center, Department of Surgery, Nijmegen, the Netherlands ^3^Amsterdam UMC, location University of Amsterdam, Department of Surgery, Amsterdam, the Netherlands
^4^Cancer Center Amsterdam, the Netherlands
^5^Erasmus University Medical Center, Department of Surgery Rotterdam, the Netherlands
^6^Radboud University Medical Center, Department of Gastroenterology, Nijmegen, the Netherlands
^7^Maastricht University Medical Center, Department of Surgery, Maastricht, the Netherlands
^8^University Medical Center Groningen, Department of Surgery, Groningen, the Netherlands
^9^Leiden University Medical Center, Department of Surgery. Leiden, the Netherlands
^10^Regional Academic Cancer Center Utrecht, Department of Surgery. Utrecht, the Netherlands

^11^St. Antonius Hospital, Department of surgery, Nieuwegein, the Netherlands
^12^Catharina Hospital, Department of Surgery, Eindhoven, the Netherlands
^13^Amsterdam UMC, location University of Amsterdam, Department of Medical Oncology, Amsterdam, the Netherlands

**Corresponding author:**

Annuska Schoorlemmer

Amsterdam UMC, Department of Surgery, Location Vumc, PK 0 Y 180,

de Boelelaan 1117,

1081 HV Amsterdam

E-mail: [a.schoorlemmer@amsterdamumc.nl](mailto:a.schoorlemmer@amsterdamumc.nl)

**Supplementary Materials - Index**

| **Supplementary Figures and Tables** |  |
| --- | --- |
| **Supplementary Figure S1** Gastrointestinal symptoms over time after pancreatectomy for pancreatic cancer, stratified for no receipt of (neo)adjuvant therapy, receipt of neoadjuvant chemo(radio)therapy, adjuvant chemotherapy or both | *page 3* |
| **Supplementary Table S1** Overview of the questions included in the items and scales for gastrointestinal symptoms, health-related quality of life and functioning | *page 4* |
| **Supplementary Table S2** Baseline characteristics patients treated with pancreatectomy for pancreatic cancer who were alive (n=780) vs those who deceased at 12 months postoperatively (n=229) | *page 6* |
| **Supplementary Table S3** Multivariable Generalized Linear Mixed Models, assessing the association of gastrointestinal symptoms over time | *page 8* |
| **Supplementary Table S4** Multivariable Generalized Linear Mixed Models, assessing the association of gastrointestinal symptoms over time restricted to patients alive at 12 months postoperatively (n=780) | *page 9* |
| **Supplementary Table S5** | *page 10* |

**Supplementary Figures and Tables**

**Supplementary Figure S1** Gastrointestinal symptoms over time after pancreatectomy for pancreatic cancer, stratified for no receipt of (neo)adjuvant therapy, receipt of neoadjuvant chemo(radio)therapy, adjuvant chemotherapy or both.

**Supplementary Table S1** Overview of the questions included in the items and scales for gastrointestinal symptoms, health-related quality of life and functioning

| Gastrointestinal symptoms | |
| --- | --- |
| EORTC QLQ-C30 | **Nausea/vomiting**  1. During the past week, have you felt nauseated?  2. During the past week, have you vomited?  **Constipation**  1. During the past week, have you been constipated?  **Diarrhea**  1. During the past week, have you had diarrhea?  **Appetite loss**  1. During the past week, have you lacked appetite? |
| EORTC QLQ-PAN-26 | **Pain**  1. During the past week, have you had abdominal discomfort?  2. During the past week, have you had back pain?  3. During the past week, did you have pain during the night?  4. During the past week, did you find it uncomfortable in certain positions (e.g. lying down)?  **Eating related problems**  1. During the past week, were you restricted in the types of food you can eat as a result of your disease or treatment?  2. During the past week, were you restricted in the amounts of food you could eat as a result of your disease or treatment?  **Cachexia**  1. During the past week, have you worried about your weight being too low?  2. During the past week, did you feel weak in your arms and legs?  **Altered bowel habit**  1. During the past week, did you have frequent bowel movements?  2. During the past week, did you feel the urge to move your bowels quickly?  **Bloated feeling (official terminology is ascites)**  1. During the past week, did you have a bloated feeling in your abdomen?  **Indigestion**  1. During the past week, have you had indigestion?  **Flatulence**  1. During the past week, were you bothered by gas (flatulence)?  **Problems with taste***  1. During the past week, did food and drink taste different from usual?  **Dry mouth***  1. During the past week, did you have a dry mouth?  ***items are officially combined in multi-item scale side-effects** |
| Health-related quality of life | |
| EORTC QLQ-C30 | **Overall quality of life**  1. How would you rate your overall health during the past week?  2. How would you rate your overall quality of life during the past week? |
| Functioning | |
| EORTC QLQ-C30 | **Physical functioning**  1. Do you have any trouble doing strenuous activities, like carrying a heavy shopping bag   or a suitcase?  2. Do you have any trouble taking a long walk?  3.Do you have any trouble taking a short walk outside of the house?  4. Do you need to stay in bed or a chair during the day?  5. Do you need help with eating, dressing, washing yourself or using the toilet?  **Role functioning**  1. During the past week, were you limited in doing either your work or other daily   activities?  2. During the past week, were you limited in pursuing your hobbies or other leisure time activities?  **Emotional functioning**  1. During the past week, did you feel tense?  2. During the past week, did you worry?  3. During the past week, did you feel irritable?  4. During the past week, did you feel depressed?  **Cognitive functioning**  1. During the past week, have you had difficulty in concentrating on things, like reading a   newspaper or watching television?  2. During the past week, have you had difficulty remembering things?  **Social functioning**  1. During the past week, has your physical condition or medical treatment interfered with your family life?  2. During the past week, has your physical condition or medical treatment interfered with your social activities? |

**Supplementary Table S2** Baseline characteristics patients treated with pancreatectomy for pancreatic cancer who were alive (n=780) vs those who deceased at 12 months postoperatively (n=229)

|  |  | **Patients alive at 12 months postoperatively** | | **Patients deceased at 12 months postoperatively** | | **P-value** |
| --- | --- | --- | --- | --- | --- | --- |
|  |  | **N** | **%** | **N** | **%** |  |
| **Age** | < 65 yrs | 333 | 42.7 | 85 | 37.1 | 0.181 |
|  | 65 - 70 yrs | 148 | 19 | 38 | 16.6 |  |
|  | 70 - 75 yrs | 159 | 20.4 | 59 | 25.8 |  |
|  | >= 75 yrs | 140 | 17.9 | 47 | 20.5 |  |
| **Sex** | Male | 417 | 53.5 | 133 | 58.1 | 0.217 |
|  | Female | 363 | 46.5 | 96 | 41.9 |  |
| **ASA classification** | ASA I | 34 | 4.4 | 9 | 3.9 | 0.81 |
|  | ASA II | 458 | 58.7 | 133 | 58.1 |  |
|  | ASA III-IV | 264 | 33.8 | 77 | 33.6 |  |
|  | Unknown | 24 | 3.1 | 10 | 4.4 |  |
| **Number of comorbidities** | No comorbidity | 411 | 52.7 | 99 | 43.2 | 0.051 |
|  | Single comorbdity | 233 | 29.9 | 75 | 32.8 |  |
|  | Two or more comorbidities | 119 | 15.3 | 49 | 21.4 |  |
|  | unknown | 17 | 2.2 | 6 | 2.6 |  |
| **Tumor location** | Head | 590 | 75.6 | 184 | 80.3 | 0.283 |
|  | Body | 51 | 6.5 | 13 | 5.7 |  |
|  | Tail | 107 | 13.7 | 21 | 9.2 |  |
|  | Other/unspecified | 32 | 4.1 | 11 | 4.8 |  |
| **cT** | cT1 | 141 | 18.1 | 30 | 13.1 | 0.145 |
|  | cT2 | 420 | 53.8 | 121 | 52.8 |  |
|  | cT3 | 82 | 10.5 | 33 | 14.4 |  |
|  | cT4 | 133 | 17.1 | 45 | 19.7 |  |
|  | cTX | 4 | 0.5 | . | . |  |
| **cN** | cN0 | 642 | 82.3 | 181 | 79 | 0.439 |
|  | cN+ | 119 | 15.3 | 43 | 18.8 |  |
|  | cNX | 19 | 2.4 | 5 | 2.2 |  |
| **Radicality** | R0 | 409 | 52.4 | 92 | 40.2 | 0.002 |
|  | R1/R2 | 361 | 46.3 | 136 | 59.4 |  |
|  | Unknown | 10 | 1.3 | 1 | 0.4 |  |
| **Type of resection** | PPPD | 234 | 30 | 64 | 27.9 | 0.206 |
|  | PD with pylorus resection | 370 | 47.4 | 119 | 52 |  |
|  | Left pancreatectomy | 150 | 19.2 | 34 | 14.8 |  |
|  | Other pancreatectomy | 26 | 3.3 | 12 | 5.2 |  |
| **Chemo(radio)therapy** | None | 77 | 9.9 | 60 | 26.2 | <0.0001 |
|  | Neoadjuvant | 199 | 25.5 | 82 | 35.8 |  |
|  | Adjuvant | 301 | 38.6 | 54 | 23.6 |  |
|  | Both | 203 | 26 | 33 | 14.4 |  |
| **Neoadjuvant chemotherapy** | No | 479 | 61.4 | 143 | 62.4 | 0.777 |
|  | Yes | 301 | 38.6 | 86 | 37.6 |  |
| **Neoadjuvant chemoradiotherapy** | No | 679 | 87.1 | 200 | 87.3 | 0.91 |
|  | Yes | 101 | 12.9 | 29 | 12.7 |  |
| **Adjuvant chemotherapy** | No | 277 | 35.5 | 142 | 62 | <0.0001 |
|  | Yes | 503 | 64.5 | 87 | 38 |  |
| **Prolonged hospital stay** | No | 600 | 76.9 | 154 | 67.2 | 0.003 |
|  | Yes | 180 | 23.1 | 75 | 32.8 |  |
| **Quality of life at baseline*** | Physical fucntioning | 81.6 ± 18.2 | | 79.1 ± 19.0 | | 0.123 |
|  | Emotional functioning | 77.5 ± 19.9 | | 77.5 ± 22.2 | | 0.979 |
|  | Role functioning | 72.9 ± 27.9 | | 68.6 ± 28.0 | | 0.089 |
|  | Cognitive functioning | 85.1 ± 18.1 | | 85.2 ± 16.8 | | 0.962 |
|  | Social functioning | 78.7 ± 24.1 | | 74.9 ± 24.5 | | 0.079 |
|  | Global quality of life | 69.8 ± 19.3 | | 69.0 ± 20.4 | | 0.656 |

PPPD=pylorus preserving pancreatoduodenectomy, PD=pancreatoduodenectomy
*Quality of life (mean ± std) at baseline was filled out by 519 patients who were alive and 160 patients who deceased during the study period

**Supplementary table S3** Multivariable Generalized Linear Mixed Models, assessing the association of gastrointestinal symptoms over time.

|  | | **Nausea/**  **vomiting** | | **Appetite**  **loss** | | **Constipation** | | **Diarrhea** | | **Pain** | **Eating related**  **problems** | | **Altered bowel**  **habits** | | **Bloated**  **Feeling** | | **Indigestion** | | **Cachexia** | | **Problems**  **with taste** | | **Dry Mouth** | | **Flatulence** |
| --- | --- | --- | --- | --- | --- | --- | --- | --- | --- | --- | --- | --- | --- | --- | --- | --- | --- | --- | --- | --- | --- | --- | --- | --- | --- |
|  | | **Beta (SE)** | | **Beta (SE)** | | **Beta (SE)** | | **Beta (SE)** | | **Beta (SE)** | **Beta (SE)** | | **Beta (SE)** | | **Beta (SE)** | | **Beta (SE)** | | **Beta (SE)** | | **Beta (SE)** | | **Beta (SE)** | | **Beta (SE)** |
| **Questionnaire** | |  | |  | |  | |  | |  |  | |  | |  | |  | |  | |  | |  | |  |
| Preoperative | | Ref. | | Ref. | | Ref. | | Ref. | | Ref. | Ref. | | Ref. | | Ref. | | Ref. | | Ref. | | Ref. | | Ref. | | Ref. |
| 0-3 mo after resection | | **3.31 (1.15)** | | **11.72 (1.58)‡** | | 0.67 (1.04) | | **12.13 (1.52)‡** | | **9.71 (1.05)†** | **18.77 (1.45)‡** | | **9 (1.32)†** | | **11.15 (1.45)‡** | | **13.38 (1.53)‡** | | **10.87 (1.21)‡** | | **7.86 (1.64)†** | | **6.03 (1.53)†** | | **8.36 (1.48)†** |
| 3-6 mo after resection | | -1.07 (1.2) | | **-3.68 (1.77)** | | -1.57 (1.14) | | **7.48 (1.62)†** | | **3.7 (1.11)** | **3.98 (1.57)** | | **9.83 (1.51)†** | | **5.24 (1.6)†** | | **7.41 (1.65)†** | | **4.8 (1.37)** | | -2.08 (1.85) | | -0.58 (1.75) | | **5.85 (1.69)†** |
| 6-9 mo after resection | | **-5.81 (1.19)†** | | **-9.53 (1.74)†** | | **-3.13 (1.14)** | | **4.04 (1.64)** | | **3.92 (1.13)** | -1.9 (1.56) | | **6.04 (1.58)†** | | 3.14 (1.63) | | **4.11 (1.68)** | | -0.8 (1.39) | | **-10 (1.91)‡** | | **-6.75 (1.72)†** | | **4.32 (1.69)** |
| 9-12 mo after resection | | **-7.68 (1.29)†** | | **-14.59 (1.83)‡** | | **-4.24 (1.23)** | | 1.48 (1.64) | | **5.01 (1.26)†** | **-3.78 (1.77)** | | 3.21 (1.67) | | **3.83 (1.77)** | | 2.83 (1.84) | | **-3.24 (1.48)** | | **-15.85 (2.05)‡** | | **-7.54 (1.89)†** | | 1.5 (1.82) |
| **Age** | |  | |  | |  | |  | |  |  | |  | |  | |  | |  | |  | |  | |  |
| <65 years | | Ref. | | Ref. | | Ref. | | Ref. | | Ref. | Ref. | | Ref. | | Ref. | | Ref. | | Ref. | | Ref. | | Ref. | | Ref. |
| 65-70 years | | -1.66 (1.18) | | 1.63 (1.79) | | -0.09 (1.22) | | **-3.83 (1.7)** | | **-3.96 (1.4)** | -1.13 (1.7) | | **-3.68 (1.73)** | | -2.77 (1.88) | | **-4.48 (1.92)** | | -0.96 (1.64) | | 0.87 (1.97) | | -1.55 (2.02) | | -2.79 (1.97) |
| 70-75 years | | **-2.46 (1.13)** | | 0.22 (1.73) | | 0.64 (1.18) | | **-3.43 (1.63)** | | -2.2 (1.33) | 0.22 (1.63) | | -0.12 (1.65) | | -2.51 (1.8) | | **-3.96 (1.83)** | | -0.54 (1.57) | | 2.02 (1.89) | | **3.96 (1.93)** | | **-3.96 (1.88)** |
| ≥75 years | | **-3.26 (1.24)** | | 2.04 (1.89) | | 1.14 (1.28) | | **-4.59 (1.79)** | | **-7.11 (1.46)†** | -0.97 (1.79) | | -2.44 (1.82) | | **-5.52 (1.97)†** | | **-7.28 (2.02)†** | | -0.26 (1.72) | | **4.17 (2.08)** | | 4.04 (2.11) | | **-4.27 (2.06)** |
| **Sex** | |  | |  | |  | |  | |  |  | |  | |  | |  | |  | |  | |  | |  |
| Male | | Ref. | | Ref. | | Ref. | | Ref. | | Ref. | Ref. | | Ref. | | Ref. | | Ref. | | Ref. | | Ref. | | Ref. | | Ref. |
| Female | | **3.7 (0.85)** | | **3.14 (1.29)** | | 0.93 (0.88) | | 1.14 (1.22) | | **2.97 (1)** | **4.01 (1.23)** | | **3.01 (1.25)** | | 1.14 (1.35) | | 2.28 (1.38) | | 0.2 (1.18) | | 2.19 (1.42) | | **5.94 (1.45)†** | | **-4.2 (1.41)** |
| **ASA classification** | |  | |  | |  | |  | |  |  | |  | |  | |  | |  | |  | |  | |  |
| ASA I | | Ref. | | Ref. | | Ref. | | Ref. | | Ref. | Ref. | | Ref. | | Ref. | | Ref. | | Ref. | | Ref. | | Ref. | | Ref. |
| ASA II | | 2.99 (2.09) | | 2.68 (3.19) | | -0.04 (2.18) | | 1.93 (3.05) | | -0.72 (2.5) | 2.15 (3.05) | | 0.36 (3.1) | | -2.13 (3.37) | | -2.09 (3.44) | | 0.17 (2.93) | | -2.52 (3.52) | | 2.1 (3.63) | | -5.18 (3.54) |
| ASA III-IV | | 4.26 (2.22) | | 1.94 (3.39) | | 0.16 (2.31) | | 3.4 (3.23) | | 0.18 (2.65) | 2.71 (3.23) | | 5.41 (3.28) | | -0.52 (3.57) | | 0.96 (3.64) | | 1.38 (3.1) | | -1.14 (3.73) | | 2.57 (3.84) | | -2.02 (3.75) |
| ASA unknown | | 6.08 (3.37) | | 7.71 (5.15) | | 0.81 (3.51) | | 1.34 (4.84) | | 0.57 (3.95) | 5.46 (4.88) | | 2.01 (4.93) | | 0.6 (5.33) | | -1.44 (5.46) | | 3.37 (4.66) | | -2.75 (5.65) | | 5.04 (5.74) | | -5.34 (5.57) |
| **Comorbidity** | |  | |  | |  | |  | |  |  | |  | |  | |  | |  | |  | |  | |  |
| No comorbidity | | Ref. | | Ref. | | Ref. | | Ref. | | Ref. | Ref. | | Ref. | | Ref. | | Ref. | | Ref. | | Ref. | | Ref. | | Ref. |
| 1 comorbidity | | -0.33 (0.99) | | -0.09 (1.5) | | -0.59 (1.02) | | 1.79 (1.42) | | 0.2 (1.17) | **-3.49 (1.42)** | | -0.61 (1.44) | | **-3.89 (1.57)** | | -2.45 (1.6) | | 0.8 (1.37) | | -0.84 (1.65) | | 3.06 (1.68) | | -1.07 (1.64) |
| ≥2 comorbidities | | 0.38 (1.31) | | 1.91 (2.01) | | 0.91 (1.37) | | 0.51 (1.9) | | 1.11 (1.55) | -0.77 (1.9) | | -2.49 (1.92) | | -1.16 (2.09) | | -2.09 (2.13) | | 1.63 (1.82) | | 0.56 (2.2) | | **7.02 (2.24)†** | | -1.92 (2.18) |
| Unknown | | 1.31 (2.73) | | -3.82 (4.19) | | 3.21 (2.84) | | -4.81 (4.09) | | -3.69 (3.27) | -5.62 (4.02) | | -6.98 (4.09) | | -6.22 (4.41) | | -4.81 (4.54) | | -2.41 (3.89) | | -8.13 (4.65) | | -6.3 (4.74) | | -5.52 (4.63) |
| **Tumor location** | |  | |  | |  | |  | |  |  | |  | |  | |  | |  | |  | |  | |  |
| Head | | Ref. | | Ref. | | Ref. | | Ref. | | Ref. | Ref. | | Ref. | | Ref. | | Ref. | | Ref. | | Ref. | | Ref. | | Ref. |
| Body | | **2.32 (2.55)** | | **4.17 (3.92)** | | 0.96 (2.66) | | 1.62 (3.73) | | 0.48 (3.07) | 0.81 (3.71) | | 0.67 (3.79) | | -4.59 (4.13) | | -0.82 (4.19) | | -2.8 (3.58) | | 3.57 (4.3) | | 4.65 (4.43) | | 0.18 (4.32) |
| Tail | | 2.61 (2.78) | | 6.47 (4.26) | | -0.56 (2.9) | | -4.55 (4.06) | | -1.61 (3.34) | 2.36 (4.05) | | 0.11 (4.12) | | -3.14 (4.48) | | 0.07 (4.57) | | -3.42 (3.9) | | 7.56 (4.69) | | 3.37 (4.82) | | 0.91 (4.69) |
| Other/unspecified | | 1.68 (2.23) | | 0.8 (3.38) | | -2.24 (2.3) | | -1.92 (3.19) | | -3.93 (2.67) | -3.04 (3.22) | | 2.89 (3.28) | | **-7.02 (3.57)** | | -2.42 (3.64) | | -2.09 (3.1) | | 3.29 (3.72) | | 5.77 (3.84) | | -3.03 (3.73) |
| **cT** | |  | |  | |  | |  | |  |  | |  | |  | |  | |  | |  | |  | |  |
| cT1 | | Ref. | | Ref. | | Ref. | | Ref. | | Ref. | Ref. | | Ref. | | Ref. | | Ref. | | Ref. | | Ref. | | Ref. | | Ref. |
| cT2 | | 1.44 (1.17) | | 2.43 (1.78) | | 0.56 (1.21) | | 1.31 (1.7) | | 2.22 (1.39) | 2.03 (1.7) | | 1.13 (1.72) | | 1.16 (1.87) | | 2.98 (1.91) | | 1.93 (1.63) | | -0.17 (1.96) | | -0.7 (2.01) | | -2.46 (1.95) |
| cT3 | | **1.58 (1.67)** | | 1.41 (2.55) | | 0.13 (1.73) | | -1.12 (2.41) | | 1.05 (1.97) | 3.69 (2.41) | | -1.66 (2.45) | | 0.4 (2.66) | | 1.64 (2.71) | | -0.15 (2.31) | | -1.72 (2.79) | | -2.27 (2.86) | | -3.69 (2.78) |
| cT4 | | 2.83 (1.56) | | 4.1 (2.37) | | -1.73 (1.61) | | **11.07 (2.22)‡** | | **4.13 (1.81)** | **5.02 (2.23)†** | | **7.77 (2.26)†** | | 4.09 (2.45) | | **10.43 (2.5)‡** | | **6.34 (2.13)†** | | -0.71 (2.59) | | -2.97 (2.64) | | 1.69 (2.55) |
| cTX | | -0.85 (6.39) | | 10.27 (9.64) | | -2.7 (6.57) | | -5.75 (9.38) | | -9.66 (7.7) | 7.21 (9.36) | | **-21.59 (9.5)^** | | -3.83 (10.26) | | -4.6 (10.6) | | -13.51 (9.06) | | 6.56 (10.77) | | -8.93 (11.12) | | -4.86 (10.77) |
| **cN** | |  | |  | |  | |  | |  |  | |  | |  | |  | |  | |  | |  | |  |
| cN0 | | Ref. | | Ref. | | Ref. | | Ref. | | Ref. | Ref. | | Ref. | | Ref. | | Ref. | | Ref. | | Ref. | | Ref. | | Ref. |
| cN+ | | **2.89 (1.18)** | | **5.84 (1.79)†** | | 1 (1.22) | | **4.67 (1.68)** | | 1.14 (1.38) | **3.33 (1.69)** | | 1.54 (1.72) | | 2.84 (1.86) | | 1.95 (1.9) | | **3.5 (1.62)** | | **4.37 (1.96)** | | 2.39 (2) | | **5.22 (1.94)†** |
| cNX | | -0.46 (2.67) | | -1.77 (4.11) | | -1.88 (2.79) | | -3.1 (3.89) | | -1.5 (3.23) | -1.24 (3.89) | | -3.78 (3.98) | | 0.23 (4.33) | | -3.23 (4.39) | | -3.53 (3.75) | | 1.9 (4.5) | | -1.49 (4.65) | | -1.74 (4.53) |
| **Radicality** | |  | |  | |  | |  | |  |  | |  | |  | |  | |  | |  | |  | |  |
| R0 | | Ref. | | Ref. | | Ref. | | Ref. | | Ref. | Ref. | | Ref. | | Ref. | | Ref. | | Ref. | | Ref. | | Ref. | | Ref. |
| R1/2 | | -0.31 (0.86) | | 0.93 (1.32) | | -0.1 (0.9) | | 2.11 (1.24) | | 0.59 (1.02) | 2.14 (1.25) | | -0.17 (1.27) | | 0.08 (1.37) | | 1.48 (1.4) | | 0.95 (1.2) | | 1.27 (1.44) | | -0.56 (1.48) | | -0.33 (1.44) |
| Unknown | | 0.82 (4.85) | | -11.36 (7.43) | | -0.74 (5.04) | | **16.86 (7.01)‡** | | **13.78 (5.74)‡** | 4.59 (7) | | 11.29 (7.13) | | 7.42 (7.75) | | 8.78 (7.86) | | 8.18 (6.7) | | -1.9 (8.12) | | 11.05 (8.29) | | 3.13 (8.07) |
| **Type of resection** | |  | |  | |  | |  | |  |  | |  | |  | |  | |  | |  | |  | |  |
| PPPD | | Ref. | | Ref. | | Ref. | | Ref. | | Ref. | Ref. | | Ref. | | Ref. | | Ref. | | Ref. | | Ref. | | Ref. | | Ref. |
| PD with pylorus resection | | **2.25 (0.98)** | | 2.09 (1.49) | | -0.17 (1.02) | | 0.98 (1.41) | | -0.46 (1.16) | 2.47 (1.41) | | 1.35 (1.43) | | 2.11 (1.56) | | 0.1 (1.59) | | 1.52 (1.36) | | 1.32 (1.64) | | 0 (1.67) | | -0.06 (1.63) |
| Left pancreatectomy | | -3.42 (2.69) | | **-8.94 (4.13)†** | | **5.74 (2.81)†** | | -4.16 (3.92) | | -0.07 (3.23) | -7.32 (3.91) | | **-12.03 (3.98)‡** | | 0.18 (4.33) | | -7.97 (4.41) | | -4.22 (3.77) | | **-9.82 (4.53)†** | | -6.09 (4.66) | | -6.72 (4.53) |
| Other pancretectomy | | -2.55 (2.75) | | -3.31 (4.22) | | 0.31 (2.86) | | 3.04 (3.97) | | 0.85 (3.2) | -5.12 (3.95) | | 5.86 (3.99) | | -2.55 (4.34) | | 2.42 (4.42) | | -0.09 (3.76) | | -0.01 (4.59) | | 0.29 (4.64) | | 2.93 (4.52) |
| **Neoadjuvant chemotherapy** |  | |  | |  | |  | |  | | |  | |  | |  | |  | |  | |  | |  | |
| No | | Ref. | | Ref. | | Ref. | | Ref. | | Ref. | Ref. | | Ref. | | Ref. | | Ref. | | Ref. | | Ref. | | Ref. | |  |
| Yes | | **-2.08 (1.03)** | | **-3.6 (1.55)** | | **-2.28 (1.04)** | | **3.3 (1.46)** | | **-4.48 (1.12)** | -2.8 (1.45) | | -0.15 (1.45) | | **-3.88 (1.54)** | | -0.51 (1.59) | | -1.28 (1.35) | | 2.77 (1.69) | | 0.04 (1.67) | | 0.91 (1.62) |
| **Neoadjuvant radiotherapy** |  | |  | |  | |  | |  | | |  | |  | |  | |  | |  | |  | |  | |
| No | | Ref. | | Ref. | | Ref. | | Ref. | | Ref. | Ref. | | Ref. | | Ref. | | Ref. | | Ref. | | Ref. | | Ref. | |  |
| Yes | | 1.96 (1.42) | | 0.61 (2.17) | | 2.47 (1.44) | | **-4.7 (2.08)** | | 1.76 (1.57) | 1.8 (2.04) | | -0.93 (2.04) | | 2.38 (2.17) | | -0.71 (2.25) | | -0.21 (1.9) | | -4.36 (2.37) | | -4.41 (2.35) | | -3.2 (2.28) |
| **Adjuvant chemotherapy** | |  | |  | |  | |  | |  |  | |  | |  | |  | |  | |  | |  | |  |
| No | | Ref. | | Ref. | | Ref. | | Ref. | | Ref. | Ref. | | Ref. | | Ref. | | Ref. | | Ref. | | Ref. | | Ref. | | Ref. |
| Yes | | 1.22 (0.99) | | -0.26 (1.48) | | -1.44 (0.97) | | -0.42 (1.43) | | **-6.81 (1.05)†** | **-3.98 (1.39)** | | -1.25 (1.34) | | **-5.3 (1.48)†** | | -2.98 (1.53) | | -0.6 (1.23) | | **7.3 (1.61)†** | | 0.73 (1.54) | | 2.3 (1.49) |
| **Prolonged hospital stay** | |  | |  | |  | |  | |  |  | |  | |  | |  | |  | |  | |  | |  |
| No | | Ref. | | Ref. | | Ref. | | Ref. | | Ref. | Ref. | | Ref. | | Ref. | | Ref. | | Ref. | | Ref. | | Ref. | | Ref. |
| Yes | | 1.28 (0.99) | | 0.9 (1.51) | | 1.27 (1.03) | | 1.53 (1.42) | | 0.8 (1.16) | 2.45 (1.43) | | 0.26 (1.44) | | -0.37 (1.57) | | 1.77 (1.6) | | 0.59 (1.36) | | 2.31 (1.65) | | **5.21 (1.68) †** | | 1.07 (1.64) |

Beta’s with standard error are presented. Analyses adjusted for age, sex, ASA, comorbidity, location of the tumor, cT, cN, radicality, type of surgery, (neo)adjuvant chemo(radio)therapy, length of hospital stay
Significant differences according to baseline are indicated in bold

PD=pancreatoduodenectomy, PRPD=pylorus preserving pancreatoduodenectomy, PRPD=pylorus resecting pancreatoduodenectomy
† small clinical relevant difference compared to baseline
‡ moderate clinical relevant difference compared to baseline

^ large clinical relevant difference compared to baseline

**Supplementary Table S4** Multivariable Generalized Linear Mixed Models, assessing the association of gastrointestinal symptoms over time restricted to patients alive at 12 months postoperatively (n=780)

| **Gastrointestinal symptom** | **Preoperative** | **0-3 mo after resection** | **3-6 mo after resection** | **6-9 mo after resection** | **9-12 mo after resection** |
| --- | --- | --- | --- | --- | --- |
| Nausea and vomiting | 14.13 (2.54) | **16.64 (2.52)** | 12.52 (2.48) | **7.82 (2.44)†** | **5.29 (2.44)†** |
| Appetite loss | 25.37 (3.93) | **36.31 (3.89)‡** | **20.02 (3.82)†** | **14.36 (3.78)‡** | **8.12 (3.77)‡** |
| Constipation | 10.46 (2.71) | 10.54 (2.69) | 8.13 (2.66) | **6.58 (2.62)** | **4.93 (2.62)** |
| Diarhoea | 14.35 (3.98) | **25.90 (3.96)‡** | **21.87 (3.91)†** | **18.40 (3.87)** | 15.67 (3.87) |
| Pain | 18.18 (3.12) | **26.46 (3.09)†** | 19.23 (3.05) | 19.31 (3.04) | 19.80 (3.06) |
| Eating related problems | 19.64 (3.82) | **38.14 (3.78)‡** | 22.69 (3.72) | 17.24 (3.69) | **14.04 (3.73)** |
| Altered bowel habits | 25.71 (4.03) | **34.67 (3.94)†** | **35.44 (3.93)†** | **31.60 (3.92)†** | 28.74 (3.95) |
| Bloated feeling | 17.67 (4.28) | **27.37 (4.23)†** | 20.37 (4.19) | 18.23 (4.18) | 18.56 (4.21) |
| Indigestion | 19.62 (4.33) | **31.51 (4.27)‡** | **25.22 (4.24)†** | 22.20 (4.21) | 20.09 (4.25) |
| Cachexia | 21.77 (3.68) | **30.36 (3.60)†** | 23.50 (3.59) | **18.47 (3.58)** | **15.46 (3.60)†** |
| Problems with taste | 32.10 (4.43) | **39.67 (4.36)†** | 28.46 (4.31) | **20.99 (4.28)‡** | **13.81 (4.31)‡** |
| Dry mouth | 33.64 (4.65) | **37.75 (4.56)** | 30.97 (4.53) | **24.86 (4.50)†** | **23.02 (4.54)‡** |
| Flatulence | 34.83 (4.56) | **43.08 (4.47)†** | **41.19 (4.47)†** | **38.67 (4.45)** | 36.15 (4.48) |

Adjusted means with standard error are presented. All gastrointestinal symptom scores range between 0-100 with a higher score referring to more problems. Analyses adjusted for age, sex, ASA, comorbidity, location of the tumor, cT, cN, radicality, type of surgery, (neo)adjuvant chemo(radio)therapy, length of hospital stay. Full models are presented in supplementary table 2.
Significant differences according to baseline are indicated in bold
† small clinical relevant difference compared to baseline
‡ moderate clinical relevant difference compared to baseline

**Supplementary table S5** The association between the number of severe gastrointestinal symptoms and Health-related Quality of life using multivariable generalized linear mixed models, restricted to patients alive at 12 months post-operatively (n=780)

| **Outcome** | **0 severe gastrointestinal symptoms** | **1 severe gastrointestinal symptom** | **2 or more severe gastrointestinal symptoms** |
| --- | --- | --- | --- |
| Global health status | 81.06 (2.59) | **75.45 (2.61)†** | **63.13 (2.56)‡** |
| Cognitive functioning | 86.49 (3.16) | **83.62 (3.17)** | **76.00 (3.14)‡** |
| Emotional functioning | 88.69 (2.97) | **86.28 (2.99)** | **78.52 (2.95)‡** |
| Physical functioning | 81.75 (2.76) | **77.67 (2.77)** | **68.71 (2.74)‡** |
| Role functioning | 79.19 (3.75) | **72.30 (3.78)†** | **55.66 (3.71)^** |
| Social functioning | 88.82 (3.19) | **84.33 (3.22)** | **69.84 (3.16)‡** |

Analyses adjusted for age, sex, ASA, comorbidity, location of the tumor, cT, cN, radicality, type of surgery, (neo)adjuvant chemo(radio)therapy, length of hospital stay
Significant differences compared to group with no severe gastrointestinal symptoms are indicated in bold
† small clinical relevant difference compared to group with no severe gastrointestinal symptoms
‡ moderate clinical relevant difference compared to group with no severe gastrointestinal symptoms
^ large clinical relevant difference compared to group with no severe gastrointestinal symptoms
